# A Retrospective Hospital-Based Observational Study on the Clinical and Radiological Profile of Paraquat Poisoning in a Tertiary Care Hospital in Northeast India

**DOI:** 10.7759/cureus.96463

**Published:** 2025-11-10

**Authors:** Subhajit Mitra, Mousumi Gogoi, Anjan J Talukdar, Jayashri Medhi, Sushmita Singha, Sangitanjan Dutta, Mintu Dewri Bharali

**Affiliations:** 1 Medicine, Gauhati Medical College and Hospital, Guwahati, IND; 2 Radiology, Gauhati Medical College and Hospital, Guwahati, IND; 3 Community Medicine, Gauhati Medical College and Hospital, Guwahati, IND

**Keywords:** free radicals, herbicide, lung fibrosis, paraquat ingestion, renal failure

## Abstract

Background

Paraquat (PQ) is a safe and effective herbicide when used as directed on the label. However, ingestion of a significant amount of the concentrate (usually with the intent of self-harm) frequently has a fatal outcome.

Method

A hospital-based retrospective study was conducted on 21 patients admitted with cases of paraquat poisoning between April 1, 2024, and March 31, 2025. Information was retrieved from secondary data that were stored in the medical registration department of Gauhati Medical College and Hospital, Guwahati, India.

Results

Our study included 21 patients, mostly females aged 21-30 years, from rural backgrounds. The most common mode of ingestion was oral, with 90% of cases being suicidal. Symptoms include nausea, vomiting, abdominal pain, oropharyngeal ulcers, respiratory distress, decreased urine, altered sensorium, leukocytosis, thrombocytopenia, transaminitis, increased serum total bilirubin, low serum albumin, and a rise in serum creatinine. All patients received ICU care and were managed with mechanical ventilation. Out of 21 patients, 19 succumbed (90.47%) to their illness. Only two patients could be revived and discharged successfully. No significant correlation was found between high serum creatinine and death.

Conclusion

We have found overall mortality to be 90.47%, with pulmonary fibrosis and acute respiratory distress syndrome (ARDS) being the primary causes of death, followed by acute kidney injury. Since there is no specific antidote for paraquat poisoning, proper and early management of the illness will help to reduce the devastating consequences of the toxic agent.

## Introduction

Paraquat (1,1′-dimethyl-4,4′-bipyridinium dichloride) is a nonselective, fast-acting herbicide widely employed for agricultural weed control due to its potent oxidative mechanism, which generates reactive oxygen species (ROS) in plants.

Weidel and Russo first synthesized Paraquat in 1882 [1]. It's herbicidal properties were not recognized until 1955 at the Imperial Chemical Industries (ICI) laboratories in Jealott's Hill, Berkshire, England [2]. Paraquat was first manufactured and sold by ICI in early 1962 under the trade name Gramoxone, which is today one of the most commonly used herbicides.

Despite its utility, paraquat is infamous for its high toxicity to humans and animals, often resulting in fatal outcomes even with minimal exposure. Paraquat poisoning is a significant public health concern, particularly in low- and middle-income countries where the chemical is readily available. Despite global efforts to regulate its use, paraquat remains accessible and continues to be implicated in intentional self-harm and accidental poisoning.

In India, where agriculture forms the economic backbone of many regions, including Assam, paraquat is readily available and often used without adequate safety measures. This widespread accessibility, coupled with limited awareness and inadequate regulation, has made paraquat poisoning a growing public health concern. The herbicide is frequently implicated in intentional self-harm, particularly among the rural and agrarian population, with several reports indicating rising incidence in tertiary care hospitals across the country [3].

Clinically, paraquat poisoning typically follows a dose-dependent course. Doses above 40 mg/kg typically cause fulminant multiorgan failure and death within 48 hours, while ingestion of 20-40 mg/kg often yields delayed pulmonary fibrosis with death in two to four weeks. The primary mechanism of paraquat poisoning in humans is redox cycling, which produces ROS. This oxidative stress causes mitochondrial malfunction, breakdown of cell membranes, and activation of inflammatory pathways including NF-κB. This results in alveolitis, pulmonary fibrosis, renal tubular necrosis, and hepatocellular injury. According to an in vivo study by Alizadeh et al. [4], paraquat-induced oxidative stress resulted in DNA strands breaking in peripheral cells and loss of cell membrane integrity. Given the many negative effects of this toxin, especially oxidative stress and DNA damage, which can significantly affect carcinogenesis, it is advised that more appropriate and less dangerous alternatives be created or introduced in place of this pesticide.

Paraquat concentrations in the lungs after consumption result in diffuse alveolitis, hemorrhage, edema, pulmonary congestion, and severe pulmonary fibrosis. In fatal cases, the lungs typically exhibit pulmonary fibrosis and pulmonary edema [5]. The lungs concentrate paraquat via the polyamine uptake system, leading to alveolar cell death, inflammation, fibrosis, and respiratory collapse [6]. Other organs, such as the kidneys, liver, heart, and gastrointestinal tract, also suffer extensive injury due to oxidative damage [7].

A retrospective study done in southern India with 55 patients reports a 72.7% hospital mortality rate with acute kidney injury being nearly universal [8]. Another retrospective study also reported that acute kidney injury is the major clinical outcome of paraquat poisoning with a mortality rate of 58.33% [9].

Diagnosis relies heavily on clinical suspicion, particularly in rural hospitals where access to confirmatory serum or urinary paraquat levels is limited. The urine dithionite test, though simple, lacks sensitivity in delayed presentations. Radiologically, chest X-ray and high-resolution computed tomography (HRCT) can reveal early interstitial changes and fibrosis, playing a crucial role in assessing pulmonary involvement and predicting outcomes. Despite these tools, the prognosis remains grim. Mortality rates in reported Indian series range from 60% to over 80%, especially in cases without timely intervention [10].

Currently, no specific antidote exists for paraquat poisoning. Management is largely supportive, including gastrointestinal decontamination with activated charcoal or Fuller's earth, renal support, antioxidants like N-acetylcysteine, and immunosuppressive therapy using cyclophosphamide and corticosteroids. Some studies suggest marginal benefit from hemoperfusion if initiated early, though evidence remains inconclusive and access to such interventions is limited in many Indian settings [11]. Immunosuppressive pulse therapy with cyclophosphamide and methylprednisolone has shown mixed results; while some meta-analyses report reduced mortality, complications such as severe immunosuppression must be carefully monitored [12].

Despite increasing case reports and scattered regional data, there is a paucity of comprehensive studies analyzing the clinical and radiological spectrum of paraquat poisoning in India, particularly in northeastern states like Assam. This gap in the literature significantly hampers the formulation of region-specific management protocols and early risk stratification tools.

Therefore, this study is undertaken to systematically evaluate the clinical and radiological profile of paraquat poisoning in a tertiary care hospital in Assam.

## Materials and methods

Study design

In this retrospective descriptive analytical study, all patients admitted to the casualty, medicine wards, and medicine ITU with alleged paraquat poisoning were evaluated from April 1, 2024, to March 31, 2025. Information was retrieved from secondary data that were stored in the medical registration department of Gauhati Medical College and Hospital. The study has been approved by the ethical committee of our college.

Inclusion and exclusion criteria

Patients above 12 years with an alleged history of paraquat poisoning were included. Those with incomplete secondary data and concurrent poisoning with chronic medical illness were excluded.

Ethical clearance

The study has been approved by the Ethical Committee of Gauhati Medical College and Hospital with certificate number 190/2007/pt-II/April-2025/34 on April 24, 2025.

Data collection

We reviewed the patient’s secondary data, which included age, gender, marital status, place of residence, route of intoxication, amount ingested, date and place of admission, length of hospital stay, symptoms and signs, and laboratory findings. Chest X-ray and HRCT thorax records, treatment received, and outcomes were also documented.

Statistical analysis

Fisher's exact test was used to determine the statistical significance (p-value) between survivor and non-survivor patients.

## Results

Twenty-one patients were included in this study based on the availability of secondary data. Of these, 13 (61.9%) were female, and eight (38.1%) were male. The female-to-male ratio was found to be 1.6:1. The average length of hospital stay was 22 ± 7 days. Most patients (52.3%) were in the 21-30-year age group, and all were from rural backgrounds. Sixteen patients (76.2%) were admitted within 24 hours of ingestion. Table 1 presents the baseline characteristics of patients with paraquat ingestion.

**Table 1:** Baseline characteristics of paraquat ingestion patients

Characteristics	Number (%) of patients
Gender	
Male	8 (38.09%)
Female	13 (61.9%)
Age (years)	
14-20	5 (23.80%)
21-30	11 (52.3%)
31-40	5 (23.80%)
Place of residence	
Rural	21 (100%)
Urban	0
Marital status	
Married	17 (80.95%)
Single	4 (19.04%)
Time of admission	
<24 hours	16 (76.19%)
>24 hours	5 (23.8%)
Nature of ingestion	
Suicidal	19 (90.47%)
Accidental	2 (9.5%)

The oral route was the most common mode of ingestion. Based on the cause, the acute poisoning patients were divided into two groups: suicidal and accidental. Paraquat ingestion was suicidal in 19 (90.47%) cases and accidental in 2 (9.5%). The exact amount of poison ingested could not be determined, as no records were available from secondary data.

The predominant symptoms following ingestion were nausea (95.2%), vomiting (95.2%), abdominal pain (66.7%), oropharyngeal ulcers (14.3%), respiratory distress (100%), decreased urine output (100%), and altered sensorium (9.5%). Leukocytosis was observed in 95.2% of cases, and thrombocytopenia was present in 14.3% on admission. Transaminitis occurred in 28.6% of patients, and increased serum total bilirubin was found in 19.0%. Most patients (38.09%) had low serum albumin levels on admission. Clinical and laboratory findings of the patients are presented in Tables 2, 3, respectively.

**Table 2:** Clinical features

Clinical and radiological features	No (%) of patients		P-value (Fisher’s exact test)
	Survivors (2)	Non-survivors (19)	
Nausea			
Yes	1 (4.76%)	19 (90.48%)	0.09
No	1 (4.76%)	0	
Vomiting			
Yes	1 (4.76%)	19 (90.48%)	0.09
No	1 (4.76%)	0	
Abdominal pain			
Yes	0	14 (66.67%)	0.1
No	2 (9.52%)	5 (23.81%)	
Oral ulcers			
Yes	1 (4.76%)	2 (9.52%)	0.2
No	1 (4.76%)	17 (80.95%)	
Respiratory distress			
Yes	2 (9.52%)	19 (90.48%)	1
No	0	0	
Decreased urine			
Yes	2 (9.52%)	19 (90.48%)	1
No	0	0	
Altered sensorium			
Yes	0	2 (9.52%)	1
No	2 (9.52%)	17 (80.95%)	

**Table 3:** Laboratory findings Leukocytosis was found in 95.2% of cases; 14.2% of cases had thrombocytopenia on admission. Transaminitis was seen in 28.57% of patients, while increased serum total bilirubin was found in 19.04% of cases. Most of them had low serum albumin on admission (38.09%). A rise in serum creatinine was seen in all cases (100%), all of which had oliguric renal failure. In our study, there was no significant correlation between high serum creatinine and death (p = 1.0). Hemodialysis was done in all patients (100%), and there was no relation between hemodialysis and mortality (p = 1.0). Chest X-ray abnormalities were noted in 71.4% of cases, showing bilateral infiltrates mainly in the mid and lower zones. HRCT findings included diffuse ground-glass opacities, interstitial thickening, and patchy consolidations. HRCT: High-resolution computed tomography.

Leukocytosis	Survivors	Non-survivors	P-value (Fisher’s exact test)
Yes	2 (9.52%)	18 (85.71%)	1.0
No	0	1 (4.76%)	
*Thrombocytopenia*			
Yes	0	3 (14.29%)	1.0
No	2 (9.52%)	16 (76.19%)	
*Transaminitis*			
Yes	1 (4.76%)	5 (23.81%)	0.5
No	1 (4.76%)	14 (66.67%)	
*Low albumin*			
Yes	1 (4.76%)	7 (33.33%)	1.0
No	1 (4.76%)	12 (57.14%)	
*Hyperbilirubinemia*			
Yes	1 (4.76%)	3 (14.29%)	0.35
No	1 (4.76%)	16 (76.19%)	
*Acute kidney injury*			
Yes	2 (9.52%)	19 (90.48%)	1.0
No	0	0	
*HRCT thorax finding*			
Yes	2 (9.52%)	19 (90.48%)	1.0
No	0	0	

A rise in serum creatinine was observed in all cases, all of which presented with oliguric renal failure. Fisher’s exact test was performed to analyze the clinical and laboratory findings between survivor and non-survivor groups. In this study, no significant correlation was found between high serum creatinine levels and death cases (p = 1.0). Hemodialysis was performed in all patients, and no association was observed between hemodialysis and mortality (p = 1.0).

Chest X-ray abnormalities were noted in 71.4% of cases, showing bilateral infiltrates mainly in the mid and lower zones. HRCT findings included diffuse ground-glass opacities, interstitial thickening, and patchy consolidations.

All 21 (100%) patients received ICU care and were managed with mechanical ventilation. Only two patients could be revived and discharged successfully. The treatment outcomes are presented in Table 4.

**Table 4:** Treatment outcome .

Hemodialysis	Survivors	Non-survivors	P-value (Fisher’s exact test)
Yes	2 (9.52%)	19 (90.48%)	1.0
No	0	0	
*ICU care*			
Yes	2 (9.52%)	19 (90.48%)	1.0
No	0	0	

Out of 21 patients, 19 (90.47%) succumbed to their illness, and two were discharged; however, follow-up data were not available.

## Discussion

A lack of precise antidotes makes paraquat very dangerous to humans and has a high case fatality rate. Due to its low cost and excellent effectiveness, it is still frequently used in many nations worldwide. Its quick inactivation upon contact with soil contributes to its low environmental toxicity. Ingestion and direct skin contact are the two ways poisoning can occur. Paraquat produces burns and rashes when it comes into direct contact with the skin. Eye contact can cause irritation, burns, corneal damage, and scarring [13].

Because paraquat is the most commonly used weedicide, poisoning from it has critical toxicological significance, particularly in people from agricultural backgrounds. In our study, 90.47% of poisonings were suicidal and 9.52% were accidental, which is similar to a study conducted in a tertiary hospital in Bangladesh [14].

The majority of the patients were in the age group of 21-30 years. The severity and mortality outcomes depend on the amount ingested, but unfortunately, it was not mentioned in the bed ticket during admission.

Common clinical symptoms in our study were nausea, vomiting, abdominal pain, respiratory distress, and decreased urine output. A study conducted by Khan et al. also showed symptoms like vomiting, abdominal pain, and throat pain [14].

Liver injury was seen in 28.57% of cases. The lungs and kidneys were the most frequently involved organs in our study, and this led to ICU referral and mechanical ventilation. Bilateral infiltrates, primarily in the mid and lower zones, were observed as chest X-ray abnormalities in 71.4% of cases. A case report on paraquat poisoning also reported diffuse bilateral coalescent opacities with diffuse alveolitis [15]. Ground-glass opacities with consolidation and honeycombing were the most important findings seen on HRCT thorax.

Hemodialysis was done in all 21 cases (100%), and no relation was found with mortality. A total of 76.19% of patients were admitted to the hospital within 24 hours of paraquat ingestion. Only two patients could be revived and discharged after successful treatment.

In comparison with other studies on paraquat poisoning in India, where a 58%-61% mortality rate has been reported [9,16], we found the overall mortality to be 90.47%, with pulmonary fibrosis and ARDS being the primary causes of death, followed by acute kidney injury. Due to the retrospective nature of the study, information was available for only 21 patients.

## Conclusions

Our study has shown a high mortality rate of 90.47%. The mortality rate could be decreased by prompt diagnosis and vigorous treatment of paraquat poisoning. Even a small dose of paraquat can be lethal, and given that there is no antidote, it is very important to take strict and immediate action. It primarily affects the kidneys and lungs. The main clinical consequence of paraquat intoxication is acute renal damage, as observed in our study. Assam, being an agricultural state, has frequent and easy access to paraquat and other herbicides. This study aims to increase awareness and help prevent the devastating consequences of paraquat ingestion.
